# A randomised placebo controlled trial of VSL#3^®^ probiotic on biomarkers of cardiovascular risk and liver injury in non-alcoholic fatty liver disease

**DOI:** 10.1186/s12876-021-01660-5

**Published:** 2021-04-01

**Authors:** Pui Lin Chong, David Laight, Richard J. Aspinall, Antony Higginson, Michael H. Cummings

**Affiliations:** 1 Academic Department of Diabetes and Endocrinology, Queen Alexandra Hospital, Portsmouth, UK; 2 School of Pharmacy and Biomedical Sciences, University of Portsmouth, Portsmouth, UK; 3 Department of Gastroenterology and Hepatology, Queen Alexandra Hospital, Portsmouth, UK; 4 Radiology Department, Queen Alexandra Hospital, Portsmouth, UK; 5 Raja Isteri Pengiran Anak Saleha Hospital, Bandar Seri Begawan, 1710 Brunei Darussalam

**Keywords:** Fatty liver, Probiotic, Biomarkers

## Abstract

**Background:**

Non-alcoholic fatty liver disease (NAFLD) is associated with increased cardiovascular risk irrespective of conventional risk factors. The role of gut-liver interaction is implicated in its development. We investigated the effects of VSL#3^®^ probiotic supplementation on biomarkers of cardiovascular risk and liver injury in patients with NAFLD.

**Methods:**

A randomised, double-blinded, placebo-controlled, proof-of-concept study was undertaken. Patients with NAFLD were randomly allocated to take 2 sachets VSL#3^®^ probiotic or placebo twice daily for 10 weeks. Measurements of endothelial function (digital photoplethysmography, sVCAM-1 and cGMP), oxidative stress (glutathione ratio and LHP), inflammation (hsCRP), insulin resistance (HOMA-IR) and liver injury [transaminases, fibrosis risk score and acoustic structure quantification (ASQ)] were undertaken before and after intervention. Difference in baseline characteristics between the treatment groups was analysed using independent *t*-test or Mann Whitney U test for non-parametric data. Independent *t*-test was used to compare the outcomes at the end of the study between the two treatment groups. Wilcoxon Signed Rank test was used to determine the difference in fibrosis risk scores before and after treatment. Spearman’s correlation was used to determine any association between cardiovascular and hepatic markers at baseline.

**Results:**

Thirty-five patients completed the study (28 males and 7 females) with a mean age of 57 ± 8 years, body mass index of 32.6 ± 5.0 kg/m^2^ and a relatively short duration of NAFLD (median duration 0.3 IQR 2.0 years). No significant difference was observed in biomarkers of cardiovascular risk and liver injury following VSL#3^®^ supplementation. Significant correlations were noted between sVCAM-1 and hsCRP (rho = 0.392, *p* = 0.01), and HOMA-IR and AST (rho = 0.489, *p* < 0.01) at baseline.

**Conclusions:**

This is the first study to evaluate the effect of VSL#3^®^ on ASQ in patients with NAFLD. VSL#3^®^ did not significantly improve markers of cardiovascular risk and liver injury in patients with NAFLD. However, the study supports an association between endothelial dysfunction and inflammation in patients with NAFLD and suggests that NAFLD is linked with insulin resistance.

*Trial registration*: ISRCTN05474560 (https://doi.org/10.1186/ISRCTN05474560) Registered 9 August 2012 (retrospectively registered).

**Supplementary Information:**

The online version contains supplementary material available at 10.1186/s12876-021-01660-5.

## Background

Non-alcoholic fatty liver disease (NAFLD) is commonly encountered with an estimated prevalence of 20–30% in Western countries [1] and 6–35% worldwide [2]. There is a strong relationship between NAFLD and insulin resistance with NAFLD regarded as the hepatic manifestation of the metabolic syndrome [3]. NAFLD is often associated with obesity [4] and type 2 diabetes mellitus (T2DM) [5].

Whilst the pathophysiology of NAFLD is complex and not fully understood, postulated mechanisms include excessive hepatic triglyceride-induced inflammatory responses and generation of reactive oxygen species (ROS) leading to liver injury [6]. More recently, gut microbiota and gut-derived endotoxinaemia have been implicated in the development of NAFLD [7]. Lipopolysaccharide (LPS) is the active component of gut-derived endotoxins which exerts metabolic and inflammatory effects on the liver. A high fat diet is associated with intestinal dysbiosis resulting in higher proportion of LPS-producing bacteria in the gut and a 2–3 fold increase in LPS concentration [8]. LPS compromises intestinal barrier integrity and increases gut permeability, facilitating translocation of endotoxins into the portal circulation [9, 10]. Within the liver, LPS activates the Toll-like receptor 4 (TLR4)-dependent pathway in Kupffer cells resulting in activation of inflammatory pathways and impairment of insulin signaling [9].

Individuals with NAFLD have increased risk of cardiovascular events and mortality independent of conventional risk factors and the metabolic syndrome [5, 11–14]. NAFLD and atherosclerosis are chronic inflammatory conditions that seem to share common pathways (endothelial dysfunction, oxidative stress and inflammation).

Probiotics are non-pathogenic live micro-organisms beneficial for gut health with associated clinical benefits such as improved lipid profile [15], reduced systolic blood pressure [15], improved insulin sensitivity [16], increased antioxidant activity [17] and decreased inflammation [18].

VSL#3^®^ is a highly concentrated probiotic product which contains 8 different strains of live freeze-dried lactic acid bacteria (streptococcus thermophilus, bifidobacterium breve, bifidobacterium longum, bifidobacterium infantis, lactobacillus acidophilus, lactobacillus plantarum, lactobacillus paracasei and lactobacillus bulgaricus). VSL#3^®^-related studies on insulin resistance [19, 20], vascular inflammation [20], oxidative stress [21], endothelial dysfunction [19] and liver injury [19, 21] are limited. Nonetheless, the gut microbiota should be considered a potential therapeutic target to treat patients with NAFLD.

## Methods

The aim of this study was to examine the effects of VSL#3^®^ probiotic supplementation on biomarkers of cardiovascular risk (insulin resistance, inflammation, endothelial function and oxidative stress) and biomarkers of liver injury in patients with NAFLD. We hypothesised that VSL#3^®^ would improve these biomarkers.

Ethical approval was granted by NRES Committee South Central—Hampshire B [Whitefriars, Lewins Mead, Bristol BS1 2NT] (ref: 11/SC/0532) and written informed consent was obtained from all eligible patients. The study was conducted at the Diabetes Centre, QAH. Recruitment began in May 2012 and ended in March 2014 when the study closed. Follow-up of the last patient was completed in January 2014.

A randomised, double blinded, placebo-controlled, proof-of-concept trial was undertaken. The primary outcome was to detect changes in biomarkers of insulin resistance, oxidative stress, endothelial dysfunction, inflammation, and liver injury. The secondary outcome was to examine whether there were any relationships between insulin resistance, oxidative stress, endothelial function, inflammation and liver transaminases at baseline in patients with NAFLD.

Patients aged 25–70 years with confirmed NAFLD (either biopsy proven or based on imaging), at least a 20% risk of a cardiovascular event over the next 10 years (Qrisk2 score) [22] and HbA1c < 86 mmol/mol (10%) were recruited from Hepatology, Diabetes and Radiology departments at Queen Alexandra Hospital (QAH); a community-based obesity clinic and Primary Care in Portsmouth, UK. Qrisk2 score was multiplied by a factor of 1.87 as existing cardiovascular risk calculators do not include NAFLD as a risk factor [14].

Those with established cardiovascular disease, decompensated liver cirrhosis, allergy or intolerance to VSL#3^®^ probiotic, chronic excess alcohol intake (> 24 g per day for men and > 16 g per day for women in the last 2 years), antibiotic treatment 4 weeks prior to the study and/or more than 3 courses of antibiotic treatment over the preceding 6 months, solid organ or bone marrow transplantation, and oral corticosteroid therapy were excluded from the study.

Patients were studied before and after a 10-week intervention period with VSL#3^®^ (Danisco Inc.) probiotic or placebo at a dose of 2 sachets twice a day [20, 21, 23]. They were randomised in a 1:1 ratio between the treatment and placebo arms by a computer-generated code using random permuted blocks of randomly varying size. Investigators and patients were blinded to the intervention throughout the study period. VSL#3^®^ and placebo were supplied by Actial Farmaceutica, Italy directly to the Clinical Trials Pharmacy Service, QAH who were responsible for packaging boxes containing identical, non-identifiable sachets of VSL#3^®^ and placebo.

Patients were fasted at least 12 h prior to their study visit. The day before their visit, foods high in fibre and starch were avoided and those on insulin therapy omitted their insulin dose(s). On the day of their visit, patients were asked not to smoke or exercise 30 min prior. Consumption of other probiotic products was prohibited during the study period.

At each visit, anthropometric data (weight and waist circumference) and blood pressure (Welch Allyn; 52,000 series) were recorded. Venous blood samples were obtained to measure fasting insulin, fasting glucose, fasting lipid profile (total cholesterol, HDL cholesterol, LDL cholesterol and triglycerides), HbA1c, fructosamine, liver enzymes (ALT and AST), albumin, INR, platelet count and high sensitivity C-reactive protein, and markers of endothelial function and oxidative stress. Lactulose hydrogen breath test (LHBT) and digital photoplethysmography were performed. A subset of patients had liver ultrasound using Acoustic Structure Quantification (ASQ) (Additional file 1).

*Lactulose hydrogen breath test* was undertaken to detect small intestinal bacterial overgrowth (SIBO). Breath samples were measured using a Micro meter H2 (Micro Medical Rochester, Kent, UK). An increase in hydrogen concentration of more than 20 parts per million from baseline within 90 min of the test and a second peak at least 15 min following the initial peak constitute a positive result [24].

*Insulin resistance* was calculated using HOMA2 (homeostasis model assessment) which provides HOMA-IR (Homeostatic Model Assessment for Insulin Resistance) estimates [25]. Three paired fasting venous glucose and insulin were taken at 5-min intervals, and their mean values were entered into the HOMA2 calculator. *Inflammation* was measured by high sensitivity C-reactive protein (hsCRP) [26] using immunonephelometry on a Siemens BN Prospec nephelometer at the Biochemical Sciences Department, St Thomas’ Hospital, London.

*Endothelial function* Digital photoplethysmography is a non-invasive technique for assessment of endothelial function. Measurements were obtained using a Micro Medical Pulse Trace (Rochester, Kent, UK). Reflective index (RI) readings were recorded before and after the administration of 400 μg sublingual glycerol trinitrate (GTN; an endothelium-independent vasodilator). Following a washout, RI readings were repeated before and after inhaled Salbutamol 400 μg (an endothelium-dependent vasodilator). The mean RI readings was used to calculate the change in RI for GTN (∆RI-GTN) and Salbutamol (∆RI-Salb) thus determining endothelium-independent and dependent vasodilator changes respectively [27].

Soluble vascular cell adhesion molecule-1 (sVCAM-1) [28] was measured using the Quantikine Human sVCAM-1 immunoassay kit from R&D Systems. Cyclic guanosine monophosphate (cGMP) [29] was analysed using the cGMP Immunoassay kit from R&D Systems.

*Oxidative stress* The glutathione ratio (GSH:GSSG) was assessed using the GSSG reductase/5,5′-dithio-bis(2-nitrobenzoic acid) re-circulating method on whole blood described by Tietze [30] and Shaik and Mehvar [31] Total antioxidant capacity (TAC) was assessed using the cupric ion reducing antioxidant capacity—bathocuproinedisulfonic acid disodium salt (CUPRAC-BCS) assay as described by Campos [32]. Quantification of lipid hydroperoxides (LHP), a direct index of oxidative stress, was measured using a method described by Ruiz et al*.* which involves a coupled glutathione peroxidase-glutathione reductase reaction [33].

*Liver injury* The NAFLD fibrosis risk score [34] and FIB4 index [35] were used as predictive models of advanced fibrosis in patients with NAFLD. Acoustic Structure Quantification is a novel high definition ultrasonographic modality that processes spatial echopatterns from scanned tissues enabling measurement of fibrous structures that reflect the ultrasound beam [36]. Two Radiologists were designated to perform ASQ liver scan (Toshiba Aplio XG, Toshiba Imaging Systems) on a subset of patients. The mode, average and standard deviation of ASQ data were generated. The mode ASQ score (expressed as C^2^m) was used to compare the degree of liver fat/fibrosis instead of the average and standard deviation as the latter values can be affected by small vessels within the liver.

### Statistical analysis

This was an exploratory proof-of-concept study examining the impact of VSL#3^®^ on cardiovascular and hepatic markers given the absence of previous data upon which to undertake a power calculation.

Data were presented as mean and standard deviation or median and inter-quartile range (IQR) for non-parametric data. Independent *t*-test (or Mann Whitney U test for non-parametric data) assessed differences in baseline characteristics between treatment groups. Study outcomes between the two treatment groups were compared using independent *t*-test. Descriptive statistics for ANCOVA were presented as mean and SD. Wilcoxon Signed Rank test was used to establish any difference in fibrosis risk scores before and after treatment. Spearman’s correlation was used to determine possible associations between markers of cardiovascular risk and hepatic liver at baseline.

Statistical software IBM SPSS Statistics 22.0 for windows (IBM Corporation 2013) was used for all analyses and all tests were performed at a 5% level of significance.

## Results

**Fig. 1:**
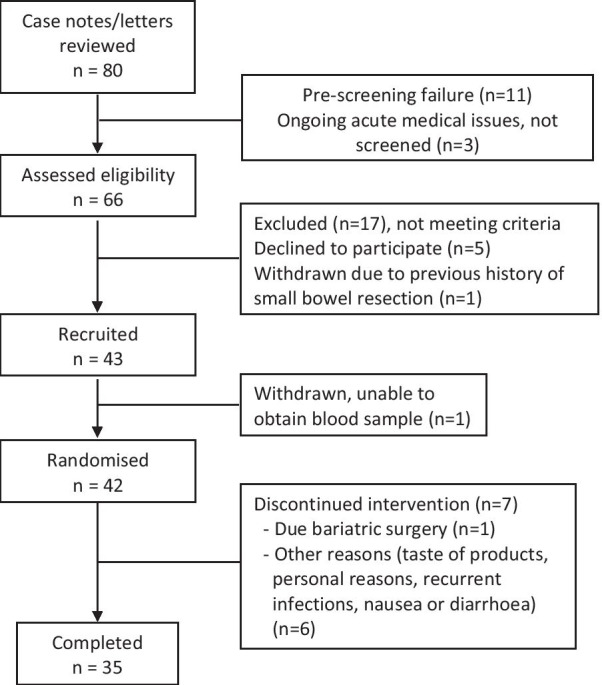
Patient recruitment

Figure 1 summaries patient recruitment. Forty-two patients participated in the study but only 35 completed. One patient was withdrawn as he was due to undergo bariatric surgery whilst 6 patients withdrew from the study for various reasons (taste of product, personal reasons, recurrent infections, nausea or diarrhoea).

Out of the 35 patients who completed the study, most were men (28 males and 7 females) with a mean age of 57 ± 8 years and a relatively short duration of NAFLD (median duration since diagnosis of 0.3 IQR 2.0 years). T2DM or impaired fasting glucose was present in 74%. Most of the patients were obese with a mean of BMI 32.6 ± 5.0 kg/m^2^ and a mean waist circumference of 111.8 ± 12.6 cm (mean for men was 110.9 ± 11.4 cm and women was 115.3 ± 17.3 cm). Baseline mean blood pressure was 134/82 ± 13/7 mmHg, total cholesterol 4.42 ± 1.15 mmol/l, HDL 1.06 ± 0.29 mmol/l, LDL 2.43 ± 1.06 mmol/l, triglycerides 2.00 ± 0.88 mmol/l and HbA1c 53 ± 14 mmol/mol.

Baseline characteristics were similar between the two treatment groups (Table 1) except HOMA-IR which was significantly lower in the VSL#3^®^-treated group (1.6 IQR 1.7 v placebo 3.0 IQR 1.8; *p* = 0.04). This difference persisted despite the exclusion of patients on insulin.

**Table 1:** Comparison of baseline characteristics between treatment groups

Measurements	Mean baseline pre-VSL#3^®^ (n = 19)	Mean baseline pre-placebo (n = 16)	*p* value
Age (y)	57 ± 8	58 ± 7	0.96
Gender (male/female)^b^	15/4	13/3	0.91
Duration NAFLD (y)^b^	0.25 IQR 2.0	1.0 IQR 1.5	0.27
No. of T2DM (or IFG)^b^	15	11	0.61
No. of smokers^b^	2	2	0.94
Alcohol (units/wk)^b^	1 IQR 8	6 IQR 8	0.10
BMI (kg/m^2^)^b^	31.2 IQR 9.1	31.9 IQR 4.0	0.57
Waist circumference (cm)	112.2 ± 14.3	111.2 ± 10.7	0.81
Systolic BP (mmHg)	133 ± 13	135 ± 12	0.56
Diastolic BP (mmHg)	82 ± 8	82 ± 7	0.91
Total cholesterol (mmol/l)	4.51 ± 1.38	4.31 ± 0.85	0.61
HDL (mmol/l)^b^	0.98 IQR 0.35	0.95 IQR 0.54	0.66
LDL (mmo/l)	2.58 ± 1.18	2.42 ± 0.70	0.64
Triglycerides (mmol/l)^b^	1.80 IQR 0.45	1.80 IQR 1.27	0.73
HbA1c (mmol/mol)^b^	54 IQR 17	47 IQR 19	0.23
HOMA-IR^b^	1.6 IQR 1.7	3.0 IQR 1.8	0.04^a^
ALT (iu/L)^b^	43 IQR 56	51 IQR 30	0.96
AST (iu/L)	40 ± 16	40 ± 15	0.99

^a^*p* < 0.05

^b^Mann Whitney U test for non-parametric data one missing value on LDL as triglyceride (> 4.5 mmol/l) was too high for LDL calculation

SIBO was present in 6 out of 35 patients (17%). No significant differences were observed in metabolic parameters and markers of insulin resistance, endothelial function, oxidative stress, inflammation and liver transaminases (ALT and AST) with VSL#3^®^ supplementation or placebo (Tables 2, 3). Even after exclusion of patients on insulin therapy, there was no significant difference in insulin resistance between the two treatment groups. Lipid hydroperoxide was excluded from analysis as levels were undetectable in all but one subject.

**Table 2:** Summary of results in patients on VSL#3^®^

Measurements	Mean pre-VSL#3^®^	Mean post-VSL#3^®^	*p* value
Systolic BP (mmHg)	133 ± 13	130 ± 11	0.53
Diastolic BP (mmHg)	82 ± 8	80 ± 7	0.42
Total cholesterol (mmol/l)	4.51 ± 1.38	4.42 ± 1.27	0.83
HDL (mmol/l)	1.07 ± 0.26	1.09 ± 0.24	0.79
LDL (mmol/l)	2.58 ± 1.18	2.56 ± 1.02	0.95
Triglycerides (mmol/l)	1.89 ± 0.57	1.91 ± 1.00	0.94
HbA1c (mmol/mol)	54 ± 12	55 ± 12	0.87
Fructosamine (μmol/l)	257 ± 44	263 ± 49	0.67
HOMA-IR	2.2 ± 1.9	2.2 ± 1.5	0.95
∆RI-GTN (%)	28 ± 6	25 ± 8	0.20
∆RI-Salb (%)	9 ± 8	8 ± 4	0.62
sVCAM-1 (ng/ml)	536 ± 305	524 ± 262	0.90
cGMP (pmol/l)	178 ± 57	159 ± 43	0.27
Blood glutathione ratio	22 ± 10	26 ± 13	0.21
TAC [mM Asc (AEAC)]	0.46 ± 0.04	0.47 ± 0.07	0.75
hsCRP (mg/l)	3.0 ± 2.5	3.9 ± 6.1	0.53
ALT (iu/l)	56 ± 31	51 ± 32	0.63
AST (iu/l)	40 ± 16	38 ± 20	0.78
Mode ASQ (C^2^m)	91 ± 14	95 ± 16	0.57

VSL#3^®^ treated group n = 19; placebo treated group n = 16. Data expressed as mean and standard deviation. Four cGMP values were not measurable (3 from placebo group and 1 from VSL#3^®^ group). HOMA-IR missing 1 value from each treatment group as insulin levels were outside the HOMA calculator range. HbA1c in the placebo group had one missing value. LDL cholesterol had missing data on 1 patient from placebo group and 1 from VSL#3^®^ group as triglycerides were > 4.5 mmol/l which preclude LDL cholesterol calculation. SBP and DBP values were missing after intervention on 1 patient in the placebo group. Nineteen patients had completed ASQ (10 in VSL#3^®^ group and 11 in placebo group). Missing data were excluded from respective analyses

**Table 3:** Summary of results in patients on placebo

Measurements	Mean pre-placebo	Mean post-placebo	p value
Systolic BP (mmHg)	135 ± 13	128 ± 17	0.19
Diastolic BP (mmHg)	82 ± 7	78 ± 11	0.27
Total cholesterol (mmol/l)	4.31 ± 0.85	4.50 ± 1.06	0.57
HDL (mmol/l)	1.05 ± 0.34	1.06 ± 0.35	0.96
LDL (mmol/l)	2.42 ± 0.70	2.50 ± 0.96	0.80
Triglycerides (mmol/l)	2.11 ± 1.15	2.39 ± 1.42	0.55
HbA1c (mmol/mol)	50 ± 15	51 ± 15	0.92
Fructosamine (μmol/l)	257 ± 53	266 ± 64	0.67
HOMA-IR	3.1 ± 1.8	3.0 ± 1.4	0.86
∆RI-GTN (%)	24 ± 7	23 ± 6	0.58
∆RI-Salb (%)	9 ± 6	9 ± 6	0.98
sVCAM-1 (ng/ml)	705 ± 423	722 ± 423	0.91
cGMP (pmol/l)	177 ± 46	177 ± 59	0.98
Blood glutathione ratio	20 ± 12	21 ± 9	0.65
TAC (mM Asc [AEAC])	0.43 ± 0.06	0.44 ± 0.07	0.62
hsCRP (mg/l)	3.2 ± 5.3	2.7 ± 2.7	0.72
ALT (iu/l)	51 ± 19	49 ± 26	0.86
AST (iu/l)	40 ± 15	41 ± 17	0.90
Mode ASQ (C^2^m)	99 ± 10	95 ± 13	0.44

VSL#3^®^ treated group n = 19; placebo treated group n = 16. Data expressed as mean and standard deviation. Four cGMP values were not measurable (3 from placebo group and 1 from VSL#3^®^ group). HOMA-IR missing 1 value from each treatment group as insulin levels were outside the HOMA calculator range. HbA1c in the placebo group had one missing value. LDL cholesterol had missing data on 1 patient from placebo group and 1 from VSL#3^®^ group as triglycerides were > 4.5 mmol/l which preclude LDL cholesterol calculation. SBP and DBP values were missing after intervention on 1 patient in the placebo group. Nineteen patients had completed ASQ (10 in VSL#3^®^ group and 11 in placebo group). Missing data were excluded from respective analyses

There was no significant change in NAFLD fibrosis risk score or FIB4-index following VSL#3^®^ treatment (Table 4). The placebo-treated group had lower post intervention mode ASQ score than the VSL#3^®^-treated group (mode ASQ score 91 C^2^m and 99 C^2^m respectively; *p* = 0.048).

**Table 4:** Effect of VSL#3^®^ on fibrosis risk scores

	Number of patients pre-VSL#3^®^			Number of patients post-VSL#3^®^			*p* value^a^
	F0–F2	Indeterminate	F3–F4	F0–F1	Indeterminate	F3–F4	
NAFLD fibrosis risk score	1	11	7	3	9	7	0.16
FIB4 index	8	7	4	8	9	2	0.41

n = 19

F0–F2 = absence of significant fibrosis

F3–F4 = presence of significant fibrosis

^a^Wilcoxon signed rank test

**Fig. 2:**
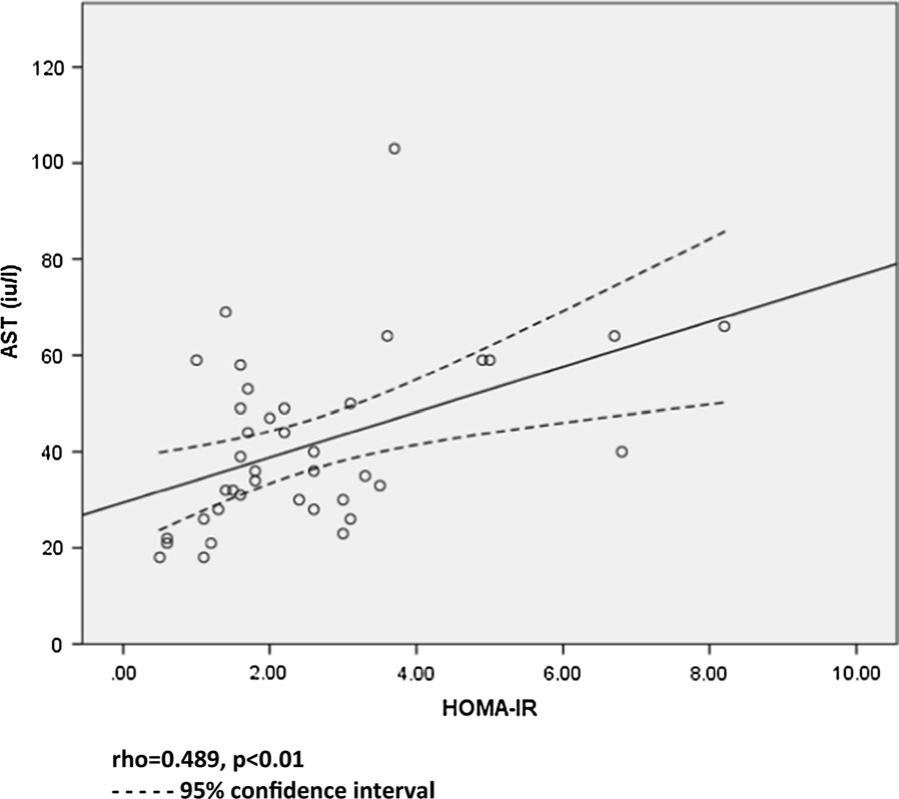
Association between baseline HOMA-IR and AST, rho = 0.489, *p* < 0.01, 95% confidence interval

Moderate correlation was observed between sVCAM-1 and hsCRP (rho = 0.392, *p* = 0.01); between hsCRP and glutathione ratio (rho = 0.325, *p* = 0.04); and between HOMA-IR and AST (rho = 0.489, *p* < 0.01) at baseline. Figure 2 shows the correlation between HOMA-IR and AST. No other associations were found.

No serious adverse events were reported in the study. In the VSL#3^®^-treated group (n = 19), 3 patients developed a urinary tract infection, 2 had bloating, 2 had nausea, 1 had genital thrush and 1 had perianal rash. In the placebo-treated group, 1 patient had diarrhoea, 1 had abdominal cramps, 1 had back pain and 1 had a traumatic toe infection.

## Discussion

In this study, VSL#3^®^ probiotic supplementation did not significantly improve insulin resistance, endothelial dysfunction, oxidative stress, inflammation or liver injury in patients with NAFLD.

Our patients with NAFLD have a higher mean baseline sVCAM-1 (714 ng/ml, range 277–1151 ng/ml) than healthy volunteers (531 ng/ml, range 341–897 ng/ml) suggesting they have underlying endothelial dysfunction. The mean baseline hsCRP (4.0 mg/l) was suggestive of a cohort at high cardiovascular risk [26]. Similar levels were reported in another study in which hsCRP was associated with a significantly higher relative risk of CVD in patients with NAFLD [13].

This is the first study to examine the impact of VSL#3^®^ on ASQ in patients with NAFLD. ASQ has not been fully validated in patients with NAFLD and the threshold that defines the severity of fatty infiltration has not been determined. Toyoda et al*.* reported strong correlation between ASQ data and grades of liver fibrosis in 148 patients with histologically proven chronic hepatitis C [36]. The median values of mode ASQ (C^2^m) of patients in the study suggest that majority of them did not have significant fibrosis [placebo group: median value of mode C^2^m was 101.0 (range 77.0–114.0) before treatment and 93.0 (83.0–123.0) after treatment; VSL#3^®^ group: 89.0 (73.0–110.0) before treatment and 96.0 (72.0–115.0) after treatment].

The majority of our patients did not have pre-existing liver biopsy to correlate ASQ data, hence it was not possible to quantify the severity of hepatic steatosis. Focal disturbance ratio (a statistical model on ASQ) can quantify liver fat in patients with NAFLD [37], however this analysis was not possible retrospectively as it required a different software version.

Several factors may have contributed to the lack of demonstrable effects of VSL#3^®^ in our cohort with NAFLD. These include a relatively small sample, and the absence of age and sex-matched controls to determine whether our cohort was at high cardiovascular risk. The difference in baseline insulin resistance between the two treatment groups despite randomisation may be partly due to the sample size.

Studies on VSL#3^®^ in patients with NAFLD are limited with the majority assessing liver-related endpoints in cirrhotic patients. Improvement in insulin sensitivity and metabolic parameters, and reduction in inflammation were demonstrated with 1 capsule of VSL#3^®^ (112.5 billion bacteria) per day over 6 weeks in overweight/obese Indian patients [20]. Reduction in markers of oxidative stress was observed with 3 months of 1800 billion bacteria daily [21]. Ultrasonographic improvement in liver fat was seen in children with NAFLD after 4 months of VSL#3^®^ [38]. The dose and duration of VSL#3^®^ vary considerably (112.5 billion bacteria to 3600 billion bacteria per day; 6 weeks to 6 months) in reported studies [20, 21, 23, 38, 39], and a 10-week intervention may be considered too short to achieve clinically meaningful results.

There were no measurements on changes in gut microbiota or endotoxin levels, which may have provided supporting evidence in the pathogenesis of NAFLD should VSL#3^®^ demonstrates modification of gut microbiota and reduction in gut-derived endotoxaemia.

Although HOMA is not validated for use in patients on insulin therapy, the use of HOMA-IR in such patients has been previously reported [40]. Re-analysis of the data excluding the 15% of patients on insulin therapy did not alter the study findings. Patients were less insulin-resistant than reported (HOMA-IR: 2.6 ± 1.8; or 2.2 ± 1.3 if those on insulin treatment were excluded) which may be related to co-existing metformin use [41].

The reproducibility of digital volume pulse as measured by digital photoplethysmography can be affected by the ambient temperature, the perfusion of patients’ index finger, patients’ position, and sudden movements (e.g. a sneeze or cough) when measurements were recorded. Maintaining similar conditions in all patients undergoing digital photoplethysmography can be challenging and considered a limitation of the study.

The moderately positive correlation between baseline hsCRP and sVCAM-1 supports an association between endothelial dysfunction and inflammation in patients with NAFLD, and this has been similarly reported in another study [42]. Paradoxically, there was a moderate positive correlation between hsCRP and blood glutathione ratio. Perhaps, there is an initial upregulation of antioxidant mechanisms to compensate for pathological states such as chronic inflammation and once these mechanisms are overwhelmed, glutathione ratio falls [43].

Insulin resistance and liver inflammation are known to be closely linked, and this has been demonstrated by the relationship between HOMA-IR and AST in our study. A similar relationship between insulin sensitivity and liver transaminases (AST and ALT) has been reported in the IRAS study [44].

The majority of VSL#3^®^ studies are based on murine models and this study provides further insight into possible effects of probiotic supplementation cardiovascular risk and liver injury in patients with NAFLD. VSL#3^®^ treatment has been shown to improve tight junction protein function [45] which is vital at maintaining intestinal epithelial barrier function and preventing translocation of gut-derived endotoxins. VSL#3^®^ inhibited hepatic JNK and NF-κB activity suggesting that treatment improved hepatic insulin sensitivity and inhibited proinflammatory pathways [46]. VSL#3^®^ also increased hepatic NKT cells which was associated with reduced TNFα expression and inhibition of IKK-β activity [47]. Decreased inflammatory mediators (TNFα, ICAM-1, RANTES and macrophage inflammatory protein-1α) within the liver was observed following treatment with VSL#3^®^, and resulted in histological improvement in liver inflammation and fibrosis [19].

## Conclusion

This study does not support the hypothesis that probiotics improve biomarkers of cardiovascular risk and liver injury in patients with NAFLD. However, a number of limitations in particular the small sample size and short duration of VSL#3^®^ treatment may have negatively influenced the study outcomes. Given modification of gut microbiota remains a potential therapeutic option in NAFLD, further studies are warranted to evaluate the benefits of probiotics in patients with NALFD.

### CONSORT guidelines

The study adheres to the CONSORT guidelines and a completed CONSORT has been submitted separately.

## Supplementary Information

**Additional file 1.** CONSORT 2010 checklist for the study.

## Data Availability

Relevant data have been presented in the main manuscript. The datasets used and/or analysed during the current study are available from the corresponding author on reasonable request.

## Abbreviations

NAFLD: Non-alcoholic fatty liver disease
sVCAM-1: Soluble vascular cell adhesion molecule-1
cGMP: Cyclic guanosine monophosphate
LHP: Lipid hydroperoxides
hsCRP: High sensitivity C-reactive protein
HOMA-IR: Homeostasis model assessment-insulin resistance
ASQ: Acoustic structure quantification
IQR: Interquartile range
T2DM: Type 2 diabetes mellitus
ROS: Reactive oxygen species
SIBO: Small intestinal bowel overgrowth
LPS: Lipopolysaccharide
TLR 4: Toll-like receptor 4
HDL: High-density lipoprotein
LDL: Low-density lipoprotein
ALT: Alanine aminotransferase
AST: Aspartate aminotransferase
LHBT: Lactulose hydrogen breath test
RI: Reflective index
GTN: Glycerol trinitrate
GSH:GSSG: Glutathione ratio
CUPRAC-BCS: Cupric ion reducing antioxidant capacity-bathocuproinedisulfonic acid disodium salt
ANCOVA: Analysis of co-variance
CVD: Cardiovascular disease
JNK: C-Jun N-terminal kinase
NF-κB: Nuclear factor kappa β
IKKß: I-κB kinase β
NKT: Natural killer T cells
RANTES: Regulated on activation, normal T cell expressed and secreted
